# Addressing the unmet need for a comprehensive lung cancer registry in Romania

**DOI:** 10.3389/fonc.2023.1211533

**Published:** 2023-06-14

**Authors:** Gheorghe-Emilian Olteanu, Cristian Iulian Oancea, Marian Catalin, Adrian Pavel Trifa, Stefan Dascalu

**Affiliations:** ^1^Center for Research and Innovation in Personalized Medicine of Respiratory Diseases. Department of Infectious Diseases, Discipline of Pulmonology, “Victor Babes” University of Medicine and Pharmacy, Timisoara, Romania; ^2^Center of Expertise for Rare Lung Diseases, Clinical Hospital of Infectious Diseases and Pneumophthisiology “Dr. Victor Babes” Timisoara, Timisoara, Romania; ^3^Research Center for Pharmaco-Toxicological Evaluations, Faculty of Pharmacy, “Victor Babes” University of Medicine and Pharmacy, Timisoara, Romania; ^4^The Discipline of Biochemistry, “Victor Babes” University of Medicine and Pharmacy, Timisoara, Romania; ^5^Center for Complex Network Science, “Victor Babes” University of Medicine and Pharmacy, Timisoara, Romania; ^6^The Discipline of Genetics, “Victor Babes” University of Medicine and Pharmacy, Timisoara, Romania; ^7^Department of Genetics, Clinical Hospital of Infectious Diseases and Pneumophthisiology “Dr. Victor Babes” Timisoara, Timisoara, Romania; ^8^Department of Genetics, Oncological Institute “Prof. Dr. Ion Chiricuta” Cluj-Napoca, Cluj-Napoca, Romania; ^9^Department of Biology, University of Oxford, Oxford, United Kingdom; ^10^Dig Worldwide Ltd, Discovery Park, Sandwich, United Kingdom

**Keywords:** cancer registry, lung cancer, Romania, epidemiological monitoring, data-driven policy-making

## Abstract

In this perspective article, we describe the vital need for a well-organized cancer registry in Romania, where lung cancer prevalence and mortality rates are alarmingly high. We discuss contributing factors such as increased use of chest X-rays and CT scans during the COVID-19 pandemic and delayed diagnoses due to limited medical care access. With the nation’s characteristically limited access to healthcare, it is plausible that the surge in acute imaging for COVID-19 has inadvertently resulted in a higher detection rate of lung cancer. This inadvertent early detection underscores the vital need for a well-organized cancer registry in Romania, where lung cancer prevalence and mortality rates are alarmingly high. Although impactful, these factors are not the primary causes of the high lung cancer cases in the country. We provide an overview of current options and propose future perspectives for epidemiological monitoring of lung cancer patients in Romania, aiming to enhance patient care, bolster research, and promote data-driven policy-making. While our primary focus is establishing a national registry for lung cancer, we address challenges, considerations, and best practices applicable to all cancer types. Through our proposed strategies and recommendations, we aim to contribute to the development and improvement of a comprehensive national cancer registry system in Romania.

## 1 Introduction

While cancer burden data and cancer research are not commonly linked with the present state of health science in Romania, both stand to benefit substantially from a well-organized and operational cancer registry. In Romania, lung cancer has reached an unparalleled prevalence and the highest mortality rate, with a multitude of factors contributing to this escalation (1–3). Among these numerous factors, two examples stand out, though they are not the primary drivers. One contributing factor is the increased utilization of chest X-rays and computed tomography (CT) scans during and after the COVID-19 pandemic. With the nation’s characteristically limited access to healthcare, it is plausible that the surge in acute imaging for COVID-19 has inadvertently resulted in a higher detection rate of lung cancer (4). These diagnoses might, under normal circumstances, have been delayed until the disease had progressed to more advanced stages or until the only viable treatment was palliative care. This inadvertent early detection underscores the vital need for a well-organized cancer registry in Romania, where lung cancer prevalence and mortality rates are alarmingly high. Another factor is the delay in diagnoses (and late-stage detection) as individuals experiencing lung cancer symptoms were unable to seek proper medical care during the pandemic. It is important to note that while these factors have a significant impact, they are not the main causes of the high number of lung cancer cases in the country.

In addition to these factors, lifestyle habits such as smoking have also played a significant role in the high incidence of lung cancer. According to the Global Adult Tobacco Survey (GATS) conducted in 2018, almost a third (30.7%) of Romanians over 15 (>5.5 million people) consumed tobacco products daily or occasionally. This prevalence was higher in men (40.4%) than in women (21.7%) (5). The estimated prevalence rate of lung cancer per 100,000 people is approximately 14.000, for which the exact epidemiology remains unknown due to the absence of national records (1–3). The only extant records are regional and do not represent the epidemiological situation on a national level (6). In this article, we explore current options and future perspectives on the epidemiological monitoring of lung cancer patients in Romania to enhance patient care, bolster research, and promote data-driven policy-making. It should be specified that our focus is on the collection of data related to primary lung tumors for the cancer registry.

Lung cancer was the leading cause of mortality of all cancers, with country-wide estimates of >10,000 deaths. The cumulative risk of dying from lung cancer was 3.45%. The 5-year estimated prevalence for lung cancer was about 14.000 cases (72 cases per 100,000 population) (1–3). However, this article does not discuss the specific modification of the mortality rate for lung cancer in Romania during the COVID-19 pandemic as more specific data or studies would be needed to accurately answer this question (7).

While our primary focus is on the need to establish a lung cancer registry, it is important to note that the challenges, considerations, and best practices discussed herein apply to all types of cancer, regardless of the organ or system involved. Furthermore, the strategies and recommendations presented can contribute to developing and improving a comprehensive national cancer registry system in Romania.

## 2 The crucial role of lung cancer registries in healthcare and research

The WHO, World Health Assembly, UN Agenda, and local authorities have implemented the Global Cancer Plan for the Prevention and Control of Non-communicable Diseases (NCD) as part of the 2030 UN Agenda for Sustainable Development Goals. This plan integrates cancer prevention and control, treatment access, palliative care, and comprehensive data collection through robust cancer registries (8, 9).

World Health Assembly Resolution 70.12 emphasizes a multi-sectorial approach to combat cancer, with cancer registries and patient surveillance being vital components. Europe’s Beating Cancer Plan, launched in 2021, represents an EU-wide strategy to reduce cancer’s impact and save at least 3 million lives by 2030. While not specifically mentioning cancer registries, it highlights the need for a Cancer Inequalities Registry (CIR) to monitor and analyze disparities in cancer outcomes (10, 11).

Cancer control (CC) lies at the core of WHO and EU initiatives, leading to national cancer control plans (NCCPs) for individual countries. NCCPs provide a framework for prioritizing and coordinating cancer prevention, detection, treatment, and care efforts. Strengthening cancer registries is a key recommendation in NCCPs, as they guide resource allocation and decision-making (12–14).

Lung cancer is a leading cause of cancer-related deaths worldwide and locally in Romania, emphasizing the need for comprehensive lung cancer registries. These registries collect patient information, offering insights into trends, disparities, and intervention areas. They play a vital role in understanding epidemiology, improving early detection, and advancing treatment options and care (15).

## 3 The benefits of lung cancer registries for healthcare and research include, but are not limited to:

Identifying risk factors. By analyzing the data from lung cancer registries, researchers can identify potential risk factors, including environmental, occupational, and lifestyle factors, which can help inform public health strategies and policies aimed at reducing lung cancer incidence. For instance, a study conducted by Raaschou-Nielsen et al. used registry data to examine the correlation between air pollution exposure and increased lung cancer incidence, highlighting the value of such registries in identifying risk factors (16).

Informing screening and early detection programs. Comprehensive lung cancer registries can provide insights into the effectiveness of screening programs and help identify populations that may benefit the most from targeted screening efforts, such as high-risk individuals or those with a family history of lung cancer.

Guiding clinical practice and treatment decisions. Lung cancer registries can inform clinicians about the best treatment approaches based on patient characteristics, tumor stage, and histology. This information can help improve patient outcomes and guide the development of personalized treatment plans.

Evaluating treatment effectiveness and outcomes. By tracking patient outcomes over time, lung cancer registries can help researchers evaluate the effectiveness of different treatment options, including surgery, radiation, chemotherapy, targeted therapies, and immunotherapy. This information can be used to inform clinical guidelines and improve patient care.

Facilitating research and collaboration. Lung cancer registries serve as a valuable resource for researchers investigating various aspects of lung cancer, including its etiology, prevention, and treatment. By providing a rich source of data, registries enable collaboration and comparison of results across different populations and settings.

Monitoring trends and disparities. Lung cancer registries can help monitor trends in incidence, prevalence, and mortality rates, as well as identify disparities in lung cancer outcomes related to socioeconomic status, race, ethnicity, or geographic location. This information can help inform targeted interventions and policies aimed at reducing these disparities.

Informing guidelines and public health interventions. For example, a study published in the International Journal of Epidemiology used registry data to examine the correlation between increased consumption of red and processed meats and the risk of colorectal cancer (17).

As the global cancer burden continues to rise, it is crucial for countries, including Romania, to establish comprehensive lung cancer registries that can inform prevention, early detection, treatment, and care strategies. Investing in these registries will not only improve lung cancer outcomes but also contribute to the broader goals of cancer control and healthcare improvement.

## 4 Current status of cancer registries in Romania

Despite the existence of clear laws that mandate cancer reporting in Romania, the current status of cancer registries is far from ideal. The last regional report dates to the period of 2010-2011 (6). This situation is paradoxical considering the following legal provisions for cancer reporting in the country:

Order of the Ministry of Health No. 219/1980: This order made cancer reporting compulsory in Romania starting January 1, 1981. It aimed to address the increasing incidence and mortality rates of cancer, which had become the second most frequent cause of morbidity and mortality after cardiovascular diseases. The order mandated that all new cancer cases be reported nominally to the Center for Calculations, Health Statistics, and Medical Documentation, directly subordinated to the Ministry of Health (18).

Order of the Ministry of Health and Family No. 871 (2002): As an update to the previous order, this order aimed to improve compliance with the cancer reporting process. Notably, it was the first Romanian Ministerial Order to involve private medical care services as suppliers of cancer case data (19).

Order of the Ministry of Public Health No. 2027/26 (November 2007): This order established a new organizational framework for cancer registration by creating regional cancer registries for each of the 8 development regions in Romania. These regional registries were to align their procedures with the standards and recommendations of the European Network of Cancer Registries (ENCR) and the International Agency for Research on Cancer (IARC) in Lyon, France. Additionally, the order emphasized the importance of adhering to data protection laws and respecting individual privacy, in line with Romanian Law No. 677/2001 and the ENCR’s Guide regarding confidentiality in population-based cancer registration in the European Union (2002) (20).

### 4.1 Some of the challenges faced by cancer registries in Romania include

Incomplete data. The coverage and completeness of cancer data in the regional registries might are limited, leading to an underestimation of the actual cancer burden in the country (6).

Limited resources. The registries face challenges related to limited financial, human, and technical resources, which affect their ability to maintain accurate and up-to-date information (6).

Fragmented data. Cancer data in Romania is fragmented, with various healthcare institutions holding different pieces of information. This can make it difficult to compile a comprehensive picture of the cancer burden in the country (6).

Lack of standardized reporting. Differences in reporting practices and data collection methods among healthcare institutions affect the quality and consistency of cancer data (6).

Limited awareness and utilization. Healthcare professionals and researchers are not fully aware of the existence of the regional registries or do not utilize the registry data to its full potential for informing cancer control strategies and research (6).

To improve the cancer registry system in Romania, efforts should be made to address these challenges and invest in the development and maintenance of accurate, comprehensive, and timely cancer registries. This will help guide cancer control efforts, inform public health policies, and support research that aims to reduce the cancer burden in the country.

## 5 Challenges and considerations for establishing a nationwide lung cancer registry

In Romania, establishing cancer registries involves overcoming various challenges and considering multiple factors in order to ensure success. Some of the key challenges and considerations include:

Collecting lifestyle data: In addition to clinical data, it’s important to collect information on the lifestyle habits of the population, particularly regarding smoking. This data can help evaluate trends in lung cancer incidence and analyze changes in risk factors. This may involve incorporating questions about smoking habits into data collection forms and ensuring that this information is accurately captured and coded in the registry.

Funding, and securing adequate funding for the establishment and long-term maintenance of a lung cancer registry is crucial. This may involve seeking support from the government, international organizations, or private funding sources.

Data collection and standardization, ensuring that data collection practices follow standardized protocols and are consistent across all institutions involved is important for producing reliable and comparable data. This may involve developing and implementing standardized data collection forms, electronic health record systems, and training programs for healthcare providers.

Data quality and completeness, ensuring high-quality and complete data is essential for accurate analysis and interpretation. In the proposed Romanian lung cancer registry, a hybrid or semiautomatic coding system could be used to ensure both accuracy and efficiency in coding cancer cases. This system would combine the strengths of both manual and automatic methods, striking a balance between accuracy and speed. The semiautomatic approach involves the initial automatic extraction of data from electronic health records (EHRs) and other sources using Natural Language Processing (NLP) algorithms. The NLP system would be designed to recognize and code key data elements, such as tumor characteristics and patient demographics, using the International Classification of Diseases for Oncology (ICD-O) and the TNM classification system (21). Despite the efficiency of automatic coding, it can sometimes overlook nuances in clinical data or misinterpret complex information. Therefore, the automatic coding process would need to be supplmented with manual review and coding by trained oncology coders. These professionals would review the automatically coded data for accuracy and completeness and manually code any complex or ambiguous data that the automatic system was unable to handle correctly. The combination of automatic and manual coding processes would thus contribute to the uniformity of coding for each case. Indeed, the automatic process ensures that the same coding standards are applied consistently across all cases, while the manual review process ensures that any errors or ambiguities in the automatically coded data are corrected. This hybrid approach would help maintain a high standard of data quality and consistency in the national cancer registry.

Interoperability with existing registries, integrating the lung cancer registry with existing cancer registries in Romania and international databases can facilitate data sharing and collaboration. This may involve adopting internationally recognized coding systems, data standards, and data exchange protocols.

Data privacy and security, protecting the privacy and security of patient data is crucial, particularly in the context of sensitive health information. This may involve implementing strict data protection policies, access controls, and encryption mechanisms to ensure compliance with Romanian Law No. 677/2001 and the ENCR’s Guide regarding confidentiality in population-based cancer registration in the European Union (2002) (18–20).

Collaboration and partnerships, establishing strong collaborations with healthcare providers, researchers, policymakers, and other stakeholders is essential for the success of the lung cancer registry. This may involve creating multidisciplinary teams, organizing workshops and conferences, and fostering a culture of cooperation and shared goals.

Public awareness and engagement, raising awareness about the importance of the lung cancer registry, and encouraging participation from patients, healthcare providers, and the general public are critical. This may involve developing public awareness campaigns, educational materials, and outreach programs.

Sustainability, ensuring the long-term sustainability of the lung cancer registry requires ongoing financial support, resource allocation, and commitment from stakeholders. This may involve developing sustainable funding models, engaging in continuous quality improvement efforts, and demonstrating the value of the registry to stakeholders through regular reporting and dissemination of findings.

## 6 Successful examples of cancer registries and lessons learned from other countries

Several countries have successfully implemented functional cancer registries, offering valuable lessons and examples that could be applied in Romania. Some of these examples and lessons learned include:

The Surveillance, Epidemiology, and End Results (SEER) Program, in the United States. SEER is a comprehensive source of cancer statistics in the United States and serves as a model for cancer registries worldwide. Key lessons from SEER include the importance of standardized data collection, rigorous data quality control, and maintaining a strong network of regional registries for comprehensive coverage (22).

The National Cancer Registry, United Kingdom. The UK National Cancer Registry collects cancer incidence and survival data from various sources, including hospital records and national screening programs. Key lessons include the benefits of electronic health record integration, coordination between multiple data sources, and the use of data linkage to provide a comprehensive view of cancer care and outcomes (23).

The Nordic Cancer Registries, Scandinavia. The Nordic countries (Denmark, Finland, Iceland, Norway, and Sweden) have long-standing, high-quality cancer registries that collaborate through the Association of the Nordic Cancer Registries (ANCR). Key lessons include the importance of international collaboration, harmonization of data collection and coding practices, and using registry data to inform public health policy and cancer prevention strategies (24).

The National Cancer Registry Programme (NCRP), India. NCRP coordinates a network of cancer registries across India, covering urban and rural populations. Key lessons include the importance of adapting registry operations to the local context, addressing disparities in cancer care, and utilizing data to inform cancer control policies and interventions (25).

The Brazilian National Cancer Institute’s Population-Based Cancer Registry (PBCR). The PBCR in Brazil collects data on cancer incidence and mortality from various sources, including hospitals and outpatient clinics. Key lessons include the importance of adapting data collection methods to the local context, investing in training and capacity building, and fostering collaboration among multiple stakeholders (26).

For the authors, the most comprehensive and successful ongoing cancer registry is The Netherlands Cancer Registry (NCR). The NCR is managed by the Netherlands Comprehensive Cancer Organisation (IKNL) and collects data on cancer incidence, prevalence, survival, and treatment from various sources, including hospitals, pathology laboratories, and radiotherapy centers (27).

### 6.1 Key lessons from the Netherlands cancer registry include

#### 6.1.1 Timeliness of data collection

The NCR is known for its prompt and up-to-date data collection, which allows for timely monitoring of cancer trends, evaluation of cancer care, and informed decision-making for policymakers and healthcare providers.

#### 6.1.2 Comprehensive data collection

The NCR collects detailed information on tumor characteristics, treatment, and follow-up care, enabling a thorough understanding of the cancer landscape and facilitating the evaluation of cancer care quality.

#### 6.1.3 Collaboration with healthcare providers

The NCR works closely with healthcare providers to collect data, offer feedback on cancer care quality, and promote improvements in cancer care delivery. This collaborative approach ensures accurate data collection and fosters a sense of shared responsibility for cancer care improvements.

#### 6.1.4 Integration with other registries and databases

The NCR is linked to other national registries and databases, such as the Pathologisch-Anatomisch Landelijk Geautomatiseerd Archief (PALGA) pathology database and the national radiotherapy registry. This integration allows for more comprehensive data coverage and enhances the potential for research and analysis (28).

#### 6.1.5 Use of data for research and quality improvement

The NCR actively supports and participates in research projects, using registry data to identify areas for improvement in cancer care and inform the development of evidence-based guidelines and policies.

#### 6.1.6 Data protection and privacy

The NCR adheres to strict data protection and privacy regulations, ensuring that patient data is securely managed and used responsibly for research and quality improvement purposes.

Drawing on the experiences of these successful cancer registries, Romania could implement best practices in data collection, standardization, data quality control, electronic health record integration, international collaboration, and stakeholder engagement to establish a comprehensive cancer registry, and of course specifically lung cancer registry (29, 30).

## 7 Existing lung cancer registries

One of the most important global lung cancer registries is the Bonnie J. Addario Lung Cancer Foundation’s (ALCF) Lung Cancer Registry (31), which is a patient-driven online platform (https://lungcancerregistry.org/) that enables lung cancer patients to share their medical data and personal experiences. Another significant registry is the Danish Lung Cancer Registry (DLCR), established by the Danish Lung Cancer Group (DLCG) with the primary goal of improving survival and the clinical management of Danish lung cancer patients. This registry also serves as a platform for lung cancer research (32).

Like the ALCF’s Lung Cancer Registry, the DLCR aims to facilitate better understanding and faster development of new treatments for lung cancer by collecting and storing patient-provided data. Researchers and medical professionals can request access to de-identified, aggregated data from both registries to analyze trends, identify gaps in care, and develop new treatment approaches.

The ALCF’s Lung Cancer Registry ensures patient privacy and data security by complying with the Health Insurance Portability and Accountability Act guidelines (33). By voluntarily contributing their information, lung cancer patients play an active role in advancing research, improving patient care, and accelerating the search for a cure. By connecting patients, researchers, and healthcare providers, both the ALCF’s Lung Cancer Registry and the Danish Lung Cancer Registry empower the lung cancer community to work together in the fight against this disease.

## 8 Applying the existing know-how in Romania

**Figure 1:**
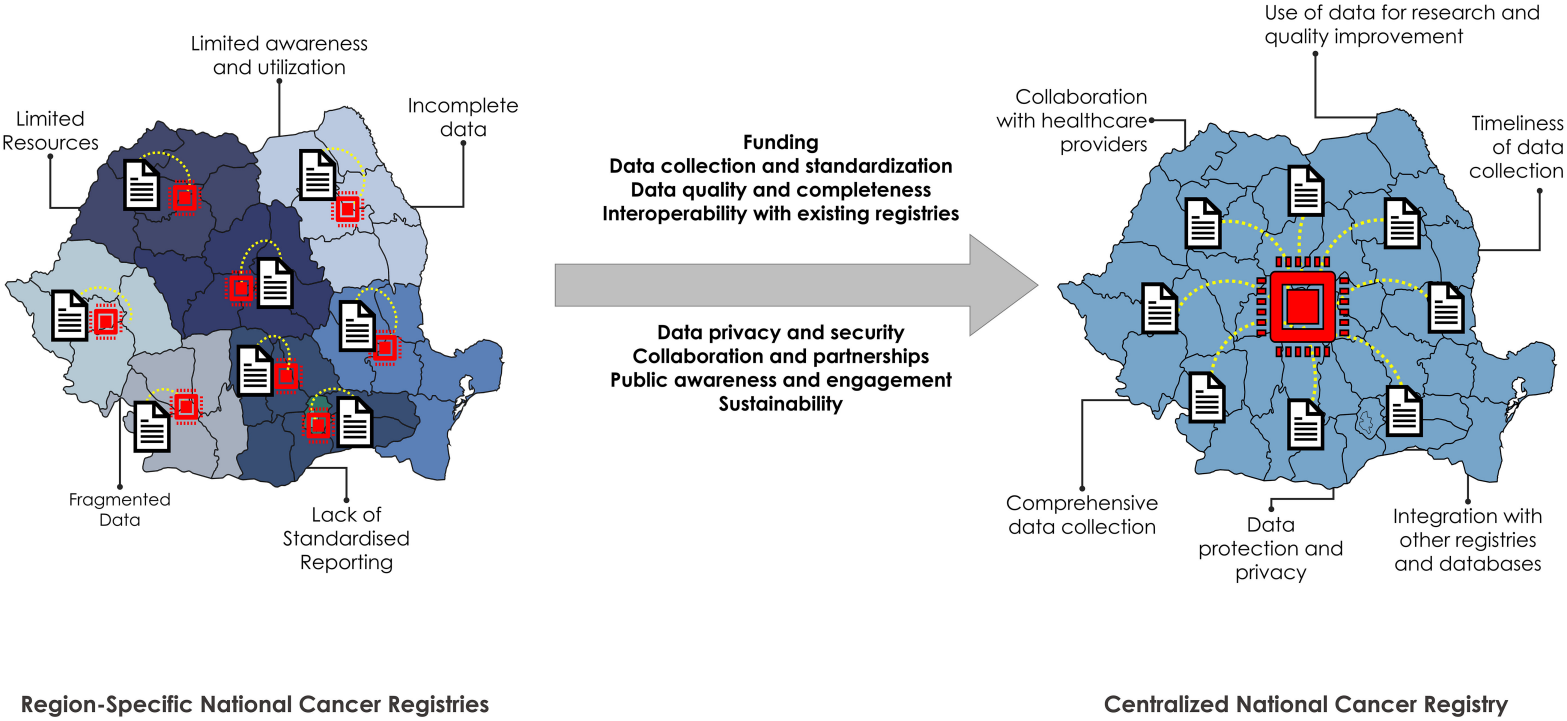
Requirements for and advantages of switching from a regional to a centralized national cancer registry in Romania. Left: current administrative distribution of the cancer registries in Romania. There are 7 macro-regions plus the capital. Current challenges and caveats are displayed next to the illustration. Right: Potential advantages of switching to a national (centralized) cancer registry in Romania.

Establishing a comprehensive and effective lung cancer registry in Romania (**Figure 1**) with the involvement of primary care physicians, including general practitioners (GPs)/family medicine (FMs) physicians:

Timely and standardized data collection: Adopting the NCR’s approach (27–30) to prompt and up-to-date data collection will enable the lung cancer registry to monitor trends, evaluate care, and inform decision-making for policymakers and healthcare providers. However, given the lack of a digital and centralized healthcare system in Romania and the low healthcare funding per capita, it is crucial to consider cost-effective and efficient methods for data collection. This may involve leveraging existing healthcare infrastructure, such as hospital records and GP/FM practices, and utilizing cost-effective digital solutions for data collection and management. The proposed semiautomatic approach, which involves the initial automatic extraction of data from EHRs and other sources using NLP algorithms, could be a viable solution for Romania. This approach would ensure both accuracy and efficiency in coding cancer cases, striking a balance between accuracy and speed.

Comprehensive data collection: Ensuring that the lung cancer registry collects detailed information on tumor characteristics, treatment, and follow-up care will facilitate a thorough understanding of the lung cancer landscape and enable the evaluation of care quality. This should also include lifestyle data, such as smoking habits, to help evaluate trends in lung cancer incidence and analyze changes in risk factors.

Collaborative relationships with healthcare providers: Working closely with oncologists, pathologists, specialized medical doctors, and general practitioners will ensure accurate data collection and foster a sense of shared responsibility for improving lung cancer care in Romania. These professionals will play a crucial role in the manual review and coding process of the semiautomatic approach, ensuring the accuracy and completeness of the data.

Integration with other registries and databases: Linking the lung cancer registry to existing national and international databases will allow for more comprehensive data coverage and enhance the potential for research and analysis.

Utilizing data for research and quality improvement: Actively supporting and participating in research projects will help identify areas for improvement in lung cancer care and inform the development of evidence-based guidelines and policies.

Robust data protection and privacy measures: Adhering to strict data protection and privacy regulations will ensure that patient data is securely managed and used responsibly for research and quality improvement purposes (34–37).

Inclusion of primary care physicians in the registry process: Engaging GPs/FMs, oncologists, pathologists, and specialized medical doctors in the lung cancer registry will enhance its effectiveness and accuracy. By providing these healthcare professionals with access to the registry and the ability to contribute data, the registry will benefit from their expertise and insights, ensuring a more comprehensive understanding of lung cancer in Romania.

To support the points raised, establishing a digital platform for real-time data registration and viewing in the lung cancer registry is essential (28–32):

- Cloud-based infrastructure
- User-friendly interface
- Role-based access control
- Data standardization and validation
- Data analytics and visualization tools
- Interoperability and data exchange
- Mobile compatibility
- Continuous updates and maintenance

By involving primary care physicians, including GPs/FMs, in the data registration and creating a digital platform that facilitates real-time data registration and viewing, Romania can establish a comprehensive and effective lung cancer registry that contributes to better understanding, prevention, and treatment of lung cancer. However, it is important to note that the success of this registry will depend on adequate funding, the commitment of healthcare professionals, and the active participation of patients. Given the current challenges in the Romanian healthcare system, it is crucial to seek innovative solutions and partnerships to overcome these obstacles and ensure the success of the lung cancer registry.

The proposed semiautomatic approach for data collection, which involves the use of NLP algorithms for initial automatic data extraction from electronic health records EHRs and other sources, could be a viable solution for Romania. This approach ensures both accuracy and efficiency in coding cancer cases, striking a balance between accuracy and speed. The NLP system is designed to recognize and code key data elements, such as tumor characteristics and patient demographics, using the ICD-O and the TNM classification system. Despite the efficiency of automatic coding, it can sometimes overlook nuances in clinical data or misinterpret complex information. Therefore, we supplement our automatic coding process with manual review and coding by trained oncology coders. These professionals review the automatically coded data for accuracy and completeness and manually code any complex or ambiguous data that the automatic system was unable to handle correctly. This hybrid approach helps us to maintain a high standard of data quality and consistency in our registry.

Furthermore, to ensure continued uniformity and accuracy in coding, we plan to conduct regular audits and provide ongoing training for our coders. This will keep our coders up to date with any changes to coding standards and will help to identify and rectify any issues with our coding process. This approach, coupled with the proposed digital platform’s features, will aid in ensuring the timeliness, uniformity, and accuracy of the data. By leveraging existing know-how, adopting innovative data collection methods, and fostering strong collaborations with healthcare providers, Romania can overcome the current challenges and establish a comprehensive and effective lung cancer registry. This will not only contribute to a better understanding, prevention, and treatment of lung cancer but also pave the way for improvements in the broader healthcare system.

## 9 Conclusion

As lung cancer has the highest mortality rate in Romania, establishing a comprehensive lung cancer registry is a crucial starting point for addressing the significant burden of this disease. This registry can serve as a pathway and master plan for creating a broader, all-encompassing cancer registry in Romania. By collecting and analyzing data on lung cancer patients, the registry can offer valuable insights for developing targeted interventions, informing policy-making, and ultimately improving patient care and outcomes across all cancer types. Despite the challenges, Romania can learn from other countries that have successfully implemented similar systems and use this lung cancer registry as a stepping stone toward a comprehensive cancer registry. Prioritizing this effort is essential in strengthening Romania’s overall strategy in the fight against cancer.

## Data availability statement

The original contributions presented in the study are included in the article/supplementary material. Further inquiries can be directed to the corresponding author.

## Author contributions

G-EO, CO, and SD jointly conceived, wrote, and edited the article. All other authors wrote and edited the article. All authors contributed to the article and approved the submitted version.
